# A TaqMan-based RT-qPCR assay for serotyping of Southern African territories (SAT) 2 strains of Foot-and-Mouth disease virus (FMDV) in Southern Africa

**DOI:** 10.1186/s13104-023-06586-7

**Published:** 2023-11-08

**Authors:** Tshephang I. Kabelo, Elliot M. Fana, Monamodi Kesamang, Joseph Hyera, Kebaneilwe Lebani

**Affiliations:** 1 Department of Biological Sciences and Biotechnology, Faculty of Science, International University of Science and Technology, Private Bag 16, Palapye, Botswana; 2 WOAH (OIE) Reference Laboratory for Foot-and-Mouth Disease, Botswana Vaccine Institute, Private Bag 0031, Gaborone, Botswana

**Keywords:** FMDV, SAT2, RT-qPCR, Serotyping

## Abstract

**Objective:**

Determining the serotype of circulating virus strains is important in implementing effective vaccination. In this study, Foot-and-Mouth Disease (FMD) Southern African territory 2 (SAT2) specific primers and TaqMan probe were designed towards rapid SAT2 detection and serotyping. The primers were tested by endpoint reverse transcription (RT) polymerase chain reaction (PCR) and quantitative PCR (RT-qPCR) using the vaccine strain SAT2035. The SAT2 serotype-specific RT-qPCR assay was compared with currently used ELISA and VP1 sequencing using Cohen’s kappa statistics.

**Results:**

The primers yielded amplicons of band size 190 bp during endpoint RT-PCR. When coupled with the probe, the primers reaction efficiency was determined to be 99% with an r^2^ value of 0.994. The results show that the SAT2 assay has comparable performance to VP1 sequencing (k = 1) and a moderate degree of agreement with ELISA (k = 0.571). The data shows that the newly designed assay could be considered for serotyping of SAT2 strains. However, for this assay to be complete there is a need to design effective SAT1 and SAT3 primers and probes that can be multiplexed to target other serotypes that co-circulate within relevant FMD endemic pools. For future implementation of the assay there is also a need to increase the number of field samples towards validation of the assay.

## Introduction

Foot-and-mouth disease (FMD) outbreaks can be very devastating to infected livestock populations and affected economies [1]. Vaccination is the most effective FMD control strategy. However, vaccination in Africa is complicated by the co-circulation of serotypes A, O, and the three Southern African Territories (SAT) serotypes of FMD virus (FMDV) in the continent. SAT serotypes are unique to Africa and are difficult to control due to their high genetic and antigenic variation [2]. These properties directly affect vaccination as each serotype has a serotype-specific vaccine. Thus, determining the serotype of circulating virus strains is important in implementing effective vaccination strategies. Currently, the main serotyping methods used in the WOAH (OIE) reference laboratory for Foot-And-Mouth disease in Botswana are enzyme-linked immunosorbert assay (ELISA) and VP1-coding region sequencing. Both methods have shortcomings. ELISAs have low sensitivity which often leads to false negatives [3] whereas VP1 sequencing can have a lengthy turn-around time. Therefore, there is a need to develop an assay which can effectively serotype FMDV strains in an accurate and timely manner. This will facilitate early control of the circulating FMDV strain/s via administration of serotype-specific vaccines to at-risk livestock populations. The aim of this study was to provide a basis for SAT-specific primers and probes which can be used for surveillance programmes and timely SAT detection during disease outbreaks. To pilot this SAT assay; SAT 2 was used as it is the frequently occurring SAT serotype in Southern Africa.

## Materials and methods

### Primer and probe design

**Fig. 1:**
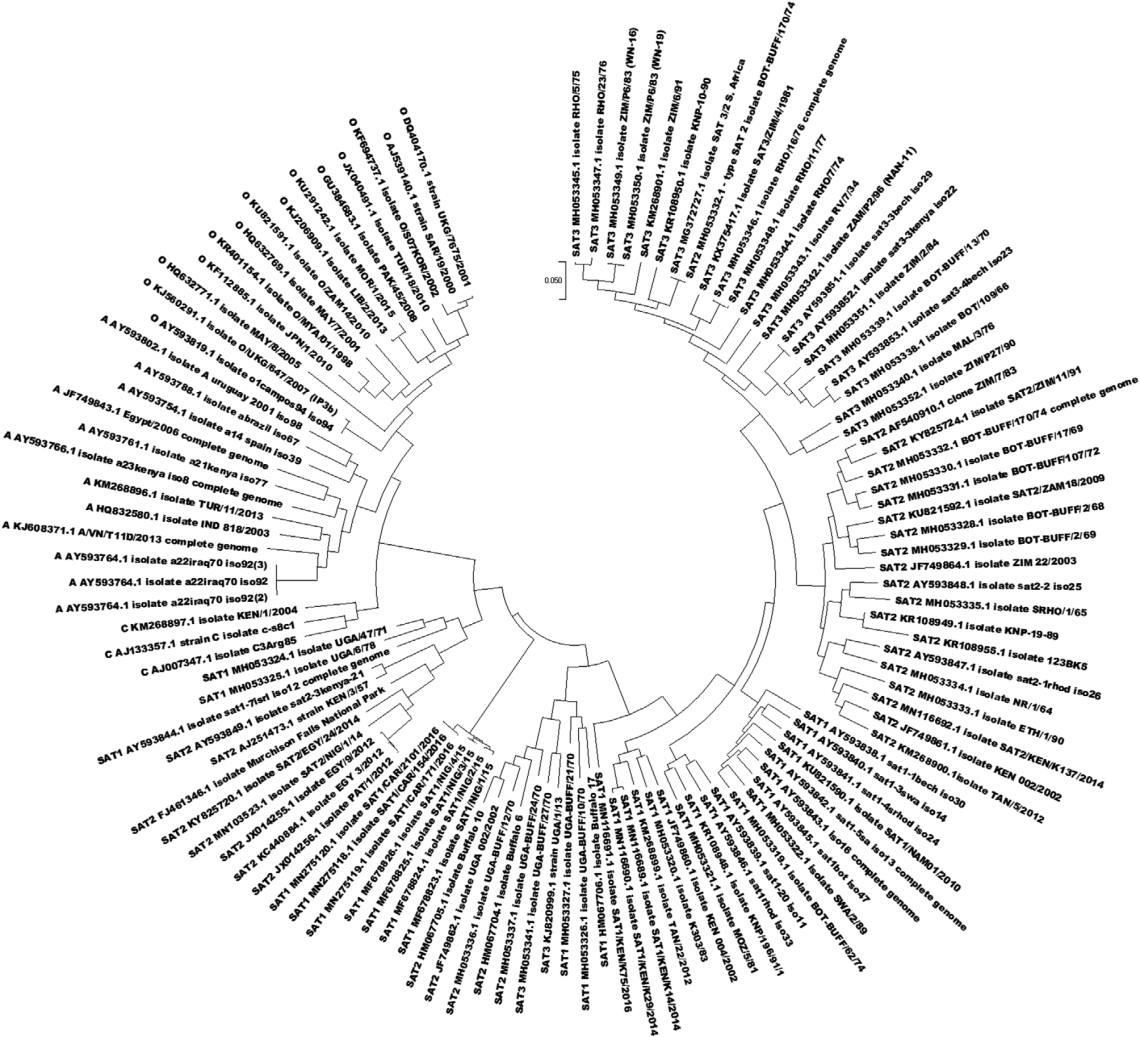
Phylogenetic tree constructed by Maximum likelihood method. This tree shows the relationship of selected NCBI sequences used for primer/probe design

SAT2-specific primers and probes were designed with a focus on the VP1-coding region of FMDV genome. Whole genome DNA sequences of the FMDV were sourced from GenBank (http://www.ncbi.nlm.nih.gov/). A total of 114 FMDV sequences belonging to SAT1 (n = 32), SAT2 (n = 32), SAT3 (n = 24), A (n = 12) and O (n = 14) serotypes (Fig. 1) were aligned against SAT2//BOT/29/98 VP1 (1D) gene reference sequence on DNA star® MegAlign pro function; MAFFT aligner (Lasergene package, DNASTAR Inc., USA). Several SNP variant loci and indels unique to SAT2 serotypes within the alignments were considered for primer design. The best scoring matches on Primer-BLAST were considered to be stable primers [4] were selected. Along with the primers, a probe sequence was designed within the amplicon 5–15 bp downstream of the forward primer. All primers and probes (Table 1) were manufactured by Inqaba Biotechnical Industries (Pretoria, South Africa). Two potential reverse primers; SAT2-VP1_R and SAT2-VP1_R2 were also synthesized.

**Table 1:** Primers and probes designed from the VP1 region of FMDV.

Primer name	Sequence (5’-3’)	Primer/probe orientation
ST2-VP1_F	TTCGTSCTBGACAGRTTCACACAYG	+
ST2-VP1_R	GACWYYGTTGTGTGARAAMACC	-
ST2-VP1_R2	CGGGGYGCVCCGTTGGGCTG	-
ST2-VP1_Probe	FAM-GTGGACCTSATGGACACCAAGGARAA-BHQ-1	+

### Virus isolates

SAT109, SAT2035, SAT306, A-Zambia and O Manisa vaccine strains were selected to test the specificity of the new SAT2 RT-qPCR primers/probes. Eight previously characterized SAT2 FMDV isolates were also selected from archival stocks in the OIE FMD RRLSA (Botswana Vaccine Institute, Botswana) repository at − 80 °C. These samples were subjected to tissue treatment and virus isolation according to the standard operating procedure for OIE laboratories [5]. Three swine vesicular disease virus (SVDV) samples were also included in this study to test the analytical specificity of the assay.

### RT-PCR

RNA was extracted manually from the selected samples using the Quick-RNA Viral kit (Zymo research) according to the manufacturer’s instructions. Individual RT-PCR reactions were set up using Luna RT-qPCR kit components (New England Biolabs,) which amounted to a total of 20 µL; containing 2 µL of RNA from the samples, 1µL of each of the 10 µM of the primers pair and the rest of the volume was made up with water. The thermal cycling parameters were set as follows; 55 °C for 10 min, 95 °C for 1 min and 40 cycles of 95 °C for 10 s and 50 − 60 °C for 1 min. After RT-PCR, an electrophoresis was run at 120 V for 80 min on a 3% agarose gel with 10 µg/mL of ethidium bromide staining solution.

### RT-qPCR

RNA was extracted manually from the selected samples using the Quick-RNA Viral kit (Zymo research) according to the manufacturer’s instructions. Individual RT-qPCR reactions were set up using Luna RT-qPCR kit components (New England Biolabs,) which amounted to a total of 20 µL; containing 2 µL of RNA from the samples, 1µL of each of the primer pairs at a concentration of 10 µM, 1µL of the serotype specific probe (at 10 µM) and the rest of the volume was made up with water. The thermal cycling parameters were set as follows; 55 °C for 10 min, 95 °C for 1 min and 40 cycles of 95 °C for 10 s and 50 − 60 °C for 1 min. The data collection point was at the final extension step.

### Standard curve

Total RNA extracted from vaccine strain SAT2035 was quantified using the Qubit RNA HS assay kit fluorometer according to the manufacturer’s instructions (Thermo Fisher Scientific, USA). A total of 2 µL of SAT2035 RNA was diluted in 18 µL nuclease free water. The RNA was serially diluted from 10^− 1^ to 10^− 6^. Each concentration was done in triplicate. RT-qPCR reactions and thermal cycling conditions were set up as described above.

### ELISA

The serotype of the eight field samples was determined by antigen typing ELISA according to a procedure described in the OIE terrestrial manual using in-house reagents [5]. A pre-determined cut off threshold was used to define a positive sample as any sample with OD < 0.3.

### VP1-coding region sequencing

VP1-coding region sequencing was performed on the eight SAT2 field samples. Briefly; the samples were subjected to endpoint RT-PCR using the above method and primers described by [6]. The amplicons obtained after RT-PCR and gel electrophoresis were excised from the gel and cleaned up using Zymoclean™ Gel DNA Recovery Kit (Zymo Research, USA). Amplicons were then sequenced bidirectionally using Sanger sequencing using primers described by (Knowles et al., 2016). The components of the reaction were from the BigDye® Terminator v3.1 cycle sequencing kit (Life Technologies, USA). The cycling parameters were as follows: 50 °C for 5 s and 60 °C for 4 min. The sequences were cleaned up using BigDye XTerminator™ Purification Kit (Applied Biosystems, California, USA) following the manufacturers protocol, for each 20 µL reaction volume the reaction plate was first pulse-centrifuged, 90 µL of Sam™ Solution was added to each well in plate and finally 20 µL of homogenised XTerminator™ solution was added. The plates were then sealed using a clear adhesive film and vortexed for 30 min and pulse-centrifuged to collect the homogenous reaction at the bottom of the plate. The cleaned sequences were then analyzed in the 3130xl genetic analyzer (Applied biosystems, USA). Chromatograms were read using Chromas 2.32 software (Technelysium Pty Ltd., Australia). The sequences were aligned using MUSCLE in MEGA X [7] and the topotypes were determined using a phylogenetic tree.

### Statistical analysis

The degree of agreement between SAT2 RT-qPCR, ELISA and VP1-coding region sequencing methods was determined by Cohen’s Kappa statistics in SPSS version 26. The precision of the assay was evaluated by calculating coefficients of variation.

## Results and discussion

In this study, a SAT 2 RT-qPCR assay was developed for the detection of FMDV SAT 2 strains circulating in Southern Africa. This is not the first account for SAT RT-qPCR development [8], however due to reduced sensitivity and specificity caused by high genetic variation within the FMDV serotypes there is need for considered re-design of primers for efficient FMDV diagnosis. Increased access to various complete VP1 gene sequences makes it possible to design efficient serotype-specific primers [9]. The SAT2 primers and probe from this study were designed from the alignment of SAT, A and O VP1 sequences (Fig. 1). Variation within VP1-coding region results in antigenically diverse serotypes. The high sequence variation within this region makes it difficult to find sites that are conserved and unique to a specific serotype [10].

**Fig. 2:**
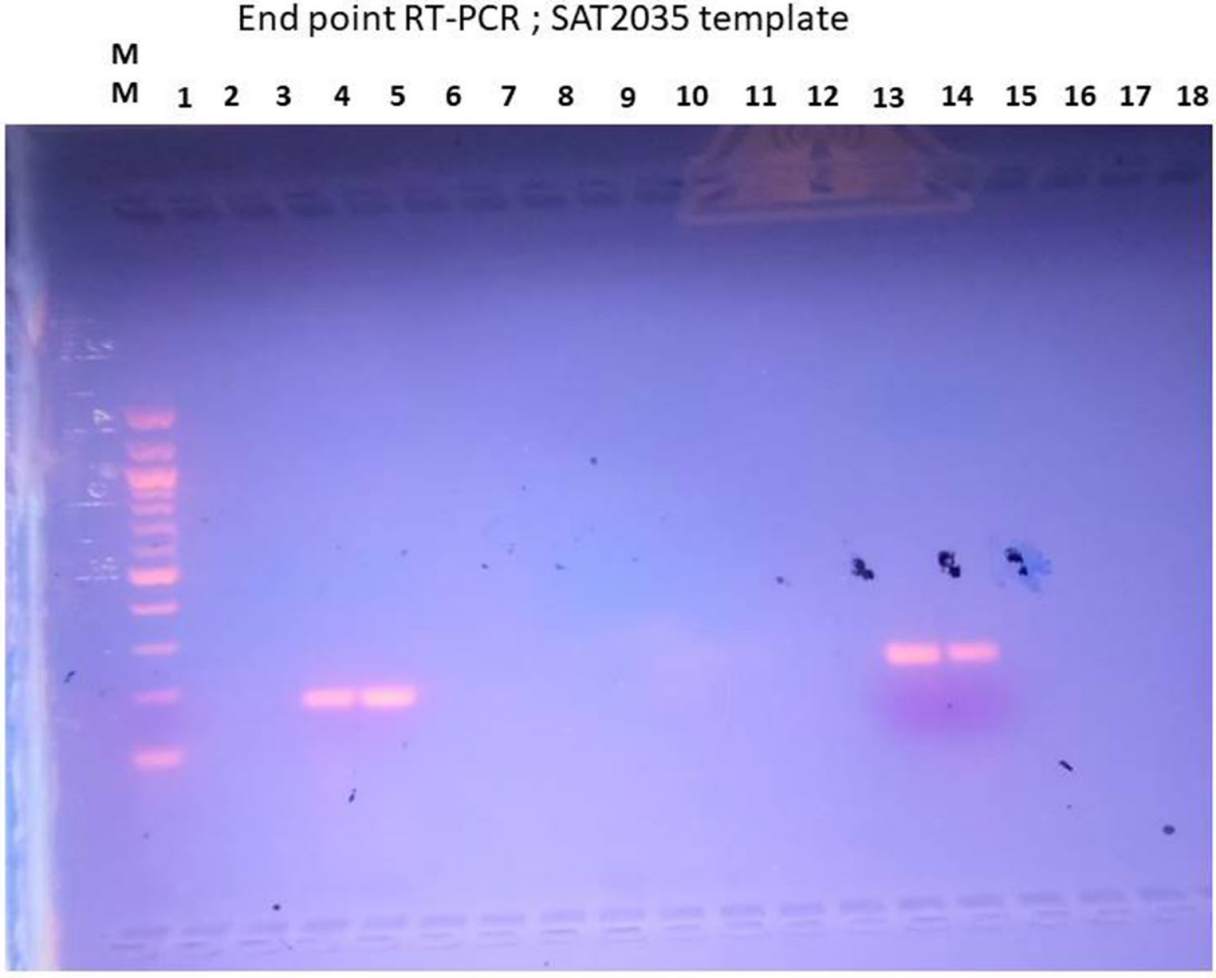
End point RT-PCR using SAT2035 RNA template. Column 1 was loaded with 100 bp molecular ladder. Columns 3 and 4 of the gel were loaded with the reactions that contained the SAT2-VP1_F and SAT2-VP1_ R2 primer set. The size of the amplicons obtained was approximately 190 bp. Column 13 and 14 contained SAT2-VP1_F and SAT2-VP1_ R primers. The size of the amplicons obtained was approximately 210 bp

One forward and two reverse primer sequences targeting regions unique to the FMDV SAT2 serotype were assessed for their ability to specifically detect the virus. To select the best performing primer set, both reverse primers were paired with the designed SAT2 forward primer to detect RNA template from SAT2035 vaccine strain. Primer sets SAT2-VP1_F and SAT2-VP1_R yielded a band of approximately 210 bp at an annealing temperature of 55 °C (Fig. 2). The primer set could not function at temperatures higher than 55 °C. Hence 55 °C was concluded to be the optimum temperature for this primer pair. At an annealing temperature of 60 °C, forward primer SAT2-VP1_F yielded a clearer band size of approximately 190 bp when paired with reverse primer SAT2-VP1_R2 (Fig. 2). SAT2-VP1_F and SAT2-VP1_R2 were thus carried forward as the selected primer set for subsequent work. The primer set was able to detect its respective vaccine strain and not produce a band for other serotypes, SVDV and the no-template control.

**Fig. 3:**
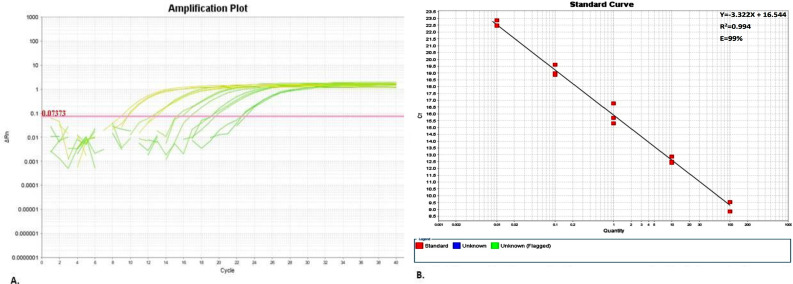
An amplification plot (**A**) and standard curve (**B**), depicting the efficiency SAT2 reactions during RT-qPCR. The reaction efficiency of SAT2 was given by a slope with an equation of y= -3.341x + 16.544, an R^2 ^value of 0.994 and a reaction efficiency of 99%

A standard curve is typically used to measure the performance of a RT-qPCR reaction by estimating its efficiency [11]. A perfectly efficient RT-qPCR should have a slope of -3.3, coefficient of determination in linear regression (r^2^) of 1 and an efficiency of 100%. To estimate the RT-qPCR efficiency of the selected SAT2 primers/probe, 10-fold serial dilutions (10^− 1^ to 10^− 6^) of quantified RNA of vaccine strains SAT2035 were performed using nuclease free water as a diluent. The concentration for the SAT2035 RNA was determined to be 72.2ng/mL before dilution. The dilutions were able to produce a standard curve with a linear correlation of y = -3.341x + 16.544 with a reaction efficiency of 99% (Fig. 3). The correlation within the reactions was given by the r^2^ value of 0.994. This result suggests that the assay has potential to have good reproducibility.

Quantitative diagnostic methods should be assessed for accuracy by testing reproducibility using technical replicates [12]. The accuracy of the selected primer set and probe was determined using the coefficient of variation. In this regard, SAT field samples were tested using RT-qPCR in triplicates. All of the SAT2 samples were positive, with CTs ranging between 10.8 and 30.8 (Table 2). The primers and probes did not yield a signal that could surpass the threshold (background fluorescence) when used to test SAT1 and SAT3 samples. These primers and probe are thus SAT2 specific. Furthermore, the no template negative controls had undetected CT values; hence the reagents used were free of any contamination. All technical repeats had coefficients of variation ranging between 0.15 and 5.62. Thus, the assay has potential to have good reproducibility.

**Table 2:** CT values, means of technical triplicates, standard deviation, and coefficient of variation for SAT 1, SAT 2 and SAT 3 field samples and SAT 109, SAT 2035, and SAT 306 vaccine strain samples

Sample name	Rep1(CT)	Rep2(CT)	Rep3(CT)	Mean	SD	%CV
SAT1/BOT/05/2014	Undetected	Undetected	Undetected	N/A	N/A	N/A
SAT1/BOT11/2015	Undetected	Undetected	Undetected	N/A	N/A	N/A
SAT1/BOT17/2014	Undetected	Undetected	Undetected	N/A	N/A	N/A
SAT1/NAM/47/2015	Undetected	Undetected	Undetected	N/A	N/A	N/A
SAT1/BOT/06/2014	Undetected	Undetected	Undetected	N/A	N/A	N/A
SAT1/NAM/43/2015	Undetected	Undetected	Undetected	N/A	N/A	N/A
SAT109	Undetected	Undetected	Undetected	N/A	N/A	N/A
SAT2/BOT/02/2017	31.01	31.94	29.54	30.83	1.21	3.93
SAT2/MOZ/06/2014	18.93	18.88	18.88	18.90	0.03	0.15
SAT2/MOZ/05/2014	14.88	13.84	13.81	14.18	0.61	4.30
SAT2/NAM/13/2017	16.53	17.00	17.11	16.88	0.31	1.82
SAT2/MOZ/03/2014	29.03	28.50	29.54	29.02	0.52	1.79
SAT2/MOZ/01/2014	22.88	24.81	23.76	23.82	0.97	4.06
SAT2/NAM/11/2017	24.86	24.88	24.87	30.83	1.21	3.93
SAT2/MAL/03/2018	20.49	20.00	21.44	20.64	0.73	3.55
SAT2035	9.89	9.80	9.77	9.82	0.06	0.64
SAT3/ZAM/07/2017	Undetected	Undetected	Undetected	N/A	N/A	N/A
SAT3/ZAM/03/2018	Undetected	Undetected	Undetected	N/A	N/A	N/A
SAT3/ZAM/08/2015	Undetected	Undetected	Undetected	N/A	N/A	N/A
SAT3/ZAM/10/2017	Undetected	Undetected	Undetected	N/A	N/A	N/A
SAT306	Undetected	Undetected	Undetected	N/A	N/A	N/A
No template control	Undetected	Undetected	Undetected	N/A	N/A	N/A

For further preliminary validation and comparison with other serotyping methods; the field samples were serotyped by antigen ELISA and VP1-coding region sequencing. The ELISA assay revealed that 6 out of 8 samples tested positive for SAT2 antigen. Two samples: SAT2/BOT/02/2017 and SAT2/NAM/11/2017 had optical density readings below 0.3 hence they were concluded to be FMDV negative, despite having produced CPE in RM cells and being positive by end-point RT-PCR. A conclusion can be made that the serotype specific antibodies used were not sensitive enough to detect all viruses within their respective serotypes.

All the field samples tested positive for the presence of SAT2 VP1-coding region. The PCR products obtained were approximately 1000 bp in size indicating successful amplification. There were no bands observed on the negative control thus there was no cross-contamination recorded. The topotypes identified for the study samples were SAT2 topotypes I and III. These topotypes typically occur in Southern Africa [2].

The degree of agreement was used to assess the ability to achieve equivalent results for the same sample using the different serotyping methods. When using Cohen kappa statistics, Cohen kappa (k) values ≤ 0 indicate no agreement and 0.01–0.20 as none to slight, 0.21–0.40 as fair, 0.41– 0.60 as moderate, 0.61–0.80 as substantial, and 0.81–1.00 as almost perfect agreement [13]. The results show that ELISA has a moderate degree of agreement with both VP1 sequencing and SAT2 RT qPCR assay (k = 0.571). These results were expected because of the documented low sensitivity of ELISAs [14]. Sequencing and SAT2 assay had a perfect degree of agreement (k = 1) because both assays detected the same number of positive samples.

In conclusion, the aim of this study was to develop an RT-qPCR assay for detecting SAT2 strains of FMDV. This study showed that the designed SAT2 assay can be a reliable system for timely SAT2 serotyping. This assay has the potential to be used in parallel with VP1 sequencing or in resource-limited settings; the assay could be used to confirm the serotype of samples missed by ELISA. The findings from this study provide a foundation for the development of a serotyping SAT assay. With more effort, a complete SAT serotyping assay incorporating SAT1 and SAT3 can be developed for fast and accurate detection of SAT serotypes in FMD-endemic pool 6 settings. The development and implementation of the potential SAT serotyping assay will facilitate timely control of SAT outbreaks via administration of appropriate serotype-specific vaccines.

### Limitations

SAT2 is the most diverse SAT serotype with 14 existing topotypes, the fidelity of the assay should be tested against various topotypes. Due to the limited availability of samples, only eight SAT 2 topotypes I and III were used. Therefore, there is a need to further test the SAT2 assay using more virus strains from different topotypes.

## Data Availability

The data that support the findings of this study have been deposited to the GenBank repository. Data is available from the authors upon reasonable request.

## List of abbreviations

CT: Cycle threshold
CPE: Cytopathic effect
CV: Coefficient of variation
E: Efficiency
ELISA: Enzyme-linked immunosorbert assay
FMD: Foot and mouth disease
FMDV: Foot and mouth disease virus
r2: Coefficient of determination in linear regression
RNA: Ribonucleic acid
K: Cohen Kappa
RT-PCR: Reverse transcription polymerase chain reaction
RT-qPCR: Reverse transcription quantitative polymerase chain reaction
SAT: Southern African Territories
SD: Standard deviation
SVD: Swine vesicular disease
VI: Virus isolation
VP1, VP2, VP3, VP4: Virus protein 1, 2, 3, 4
